# Premature mortality analysis of 52,000 deceased cats and dogs exposes socioeconomic disparities

**DOI:** 10.1038/s41598-024-77385-8

**Published:** 2024-11-20

**Authors:** Sean Farrell, Katharine Anderson, Peter-John Mäntylä Noble, Noura Al Moubayed

**Affiliations:** 1 Department of Computer Science, Durham University, Durham, UK; 2 Dogs Trust, London, UK; 3 Institute of Infection, Veterinary and Ecological Sciences, University of Liverpool, Liverpool, UK; 4 Evergreen Life Ltd, Manchester, UK

**Keywords:** Data processing, Risk factors, Machine learning, Computer science

## Abstract

Monitoring mortality rates offers crucial insights into public health by uncovering the hidden impacts of diseases, identifying emerging trends, optimising resource allocation, and informing effective policy decisions. Here, we present a novel approach to analysing premature mortality in companion animals, utilising data from 28,159 deceased dogs and 24,006 deceased cats across the United Kingdom. By employing PetBERT-ICD, an automated large language model (LLM) based *International Classification of Disease 11* syndromic classifier, we reveal critical insights into the causes and patterns of premature deaths. Our findings highlight the significant impact of behavioural conditions on premature euthanasia in dogs, particularly in ages one to six. We also identify a 19% increased risk of premature mortality in brachycephalic dog breeds, raising important animal welfare concerns. Our research establishes a strong correlation between socioeconomic status and premature mortality in cats and dogs. Areas with the lowest Index of Multiple Deprivation (IMD) scores show nearly a 50% reduction in the risk of premature mortality across cats and dogs, underscoring the powerful impact that socioeconomic factors can have on pet health and longevity. This research underscores the necessity of examining the socioeconomic disparities affecting animal health outcomes. By addressing these inequities, we can better safeguard the well-being of our companion animals.

## Introduction

Monitoring mortality rates within a population is crucial for assessing overall public health and uncovering social inequalities that influence life expectancy and quality of life^1,2^. It is well established within human medicine that there exists a link between increasing life expectancy within regions of increased economic wealth^3–5^. This relationship between economic wealth and life expectancy in humans raises questions about the health and longevity of companion animals in similar affluent regions. In human medicine, many methodologies exist to assess mortality, such as the ‘Disability-Adjusted Life Years (DALY)’^6^, ‘Quality-Adjusted Life Year (QALY)’^7^ and ‘Healthy Adjusted Life Expectancy (HALE)’ metrics, all frequently appearing within the World Health Organisation global disease burden reports^8^. Metrics can reflect the social, economic and environmental conditions individuals live and grow in and form a basis for comparing healthcare inequality and tracking global improvements. Through tracking across different regions and groups, public health officials and researchers can monitor the impact of initiatives and goals to improve standards and identify trends, gaps, and priorities for improving population health. Nevertheless, national mortality rates for companion animals are not subject to regular monitoring.

Understanding companion animal longevity could support the identification of factors causing premature death and develop systems to mitigate this. It may also reflect the impact of associated health characteristics, such as socioeconomic conditions, environmental quality, lifestyle choices, access to health care and disease prevention. The surveillance of electronic health records (EHR) collected from primary-care veterinary practices represents a valuable means to gain insights into companion animals’ current population health status. Initiatives such as the Small Animal Veterinary Surveillance Network (SAVSNET) and VetCompass have played a pivotal role in establishing accessible, real-time, first-opinion clinical EHRs on a national scale in the United Kingdom (UK)^9,10^. Despite their potential, harnessing the total utility of first-opinion veterinary EHRs on a large scale is challenging. While advantageous for researchers, implementing disease coding frameworks in clinical practice is plagued with poor compliance and impractical for everyday use. Previous studies have underscored records annotated by clinicians as part of their routine responsibilities as being particularly susceptible to inaccuracies and omissions^11,12^. Adopting an unstructured, free-text format in veterinary EHRs while affording clinicians greater linguistic flexibility presents challenges in developing automated systems^13,14^. In response to these challenges, a pressing need exists to establish fixed, tabular data points for clinical events that do not impose additional complexity on clinicians’ responsibilities whilst facilitating downstream data analysis.

The study of mortality rates in companion animals is complex, primarily due to the extensive breed variation and the diverse conditions associated with each breed. A critical factor contributing to this complexity is the cephalic index, which quantifies the ratio between the width and length of an animal’s cranium. Companion animal breeds are categorised into brachycephalic (‘short-headed’), mesaticephalic (‘middle-headed’), and dolichocephalic (‘long-headed’) based on this index. Brachycephalic breeds, such as Bulldogs and Pugs, have increasingly become a welfare concern^15–17^. Prior research has highlighted the numerous conditions prevalent among brachycephalic dogs, including heightened susceptibility to respiratory issues^18–21^, ocular diseases^22,23^, and digestive disorders^21,24^. These health challenges significantly impact their longevity, resulting in a median life expectancy of 8.6 years for brachycephalic breeds, markedly lower than the 12.7 years observed in non-brachycephalic breeds^25^. Understanding these breed-specific health issues is essential for improving welfare standards and lifespan in companion animals, illustrating the need for continued research and targeted interventions.

In this study, we present a large language model-based approach to identifying animals that have been declared deceased. We analyse a dataset comprising 28,159 deceased dogs and 24,006 deceased cats, exploring their lifetime histories from a broader dataset of approximately 143,000 dog and 93,000 cat records. Utilising PetBERT, we reveal the leading causes of lost years of life across different age groups around the International Classification of Disease 11 (ICD-11) chapter framework^26,27^. We establish a breed-specific premature longevity threshold to investigate risk factors associated with premature death in dogs and cats. By defining these thresholds, we were able to assess which phenotypic and demographic features are indicative of an increased likelihood of an animal dying before reaching its breed-specific premature longevity threshold. Finally, we discuss the impact of socioeconomic factors on premature mortality among the animal population within the UK. Through this automated record annotation and analysis, our study provides valuable insights into the factors affecting pet longevity and the potential interventions that could mitigate premature death.

## Results

### Data extraction

The SAVSNET dataset contains 9,281,287 veterinary consultation records from first opinion practices across the UK, spanning a decade from March 2014 to March 2024. In assuring data quality, records with missing values for age, owner postcode, sex, and breed were excluded. The resulting cleaned dataset retained all 9,213,720 consultations. This study focused on canine and feline patients, totalling 5,772,469 and 2,241,767 records, respectively. A BERT-based language model was fine-tuned as a binary sequence classification model to identify consultations where the attending clinician declared a death within the clinical notes. The model was trained on a dataset of 400 professionally annotated records and evaluated on a separate test set of 200 records. A practising clinician curated both datasets. The model demonstrated high performance with a precision of 0.995 and a recall of 1.000 on the test set. We then applied this model to the entire SAVSNET dataset.

### Data exploration

Among the total number of animals identified to have died, 28,159 were dogs and 24,006 were cats. For the dogs, 14,059 (49.9%) were females, and 14,100 were males, with 17,690 (63.1%) being neutered. The overall mean age for dogs at the time of death was 12.55 years, with a median age of 13.05 years. For the cats, 12,964 (54.0%) were females, and 11,042 were males, with 18,706 (77.9%) being neutered. The overall mean age for cats was 14.44 years, with a median age of 15.22 years. To determine the approximate proportion of animals that were euthanised versus those that died of natural causes, a regular expression search term and manual readings were conducted on the final EHR narrative. For cats, euthanasia accounted for 23,910 cases (99.6%), with 48 cases (0.2%) being identified as having either arrived deceased or deaths occurring within the consultation room, and 48 cases (0.2%) classified as indeterminable. Similarly, for dogs, euthanasia accounted for 28,046 cases (99.6%), while already deceased or deaths occurring within of the consultation room were recorded in 28 cases (0.1%), and 85 cases (0.3%) were considered indeterminable.

**Table 1:** Summary of median age at death, breed-specific premature longevity thresholds, total count (N), and Cephalic index for dog breeds within the dataset.

Breed	Premature threshold (years)	Median age (years)	n	Cephalic index
Airedale Terrier	9.80	11.73 (11.53–11.93)	23	Dolichocephalic
Akita	8.26	10.00 (9.72–10.28)	29	Mesaticephalic
Alaskan Malamute	9.34	11.11 (10.98–11.24)	49	Mesaticephalic
American Bulldog	8.27	9.95 (9.73–10.17)	65	Brachycephalic
Basset Hound	10.19	12.17 (11.99–12.35)	81	Dolichocephalic
Beagle	10.01	11.95 (11.78–12.12)	133	Mesaticephalic
Bearded Collie	11.41	13.62 (13.43–13.81)	45	Mesaticephalic
Bedlington Terrier	10.46	12.53 (12.31–12.75)	45	Dolichocephalic
Belgian Shepherd	10.03	11.98 (11.80–12.16)	18	Mesaticephalic
Bernese Mountain	6.92	8.30 (8.14–8.46)	20	Mesaticephalic
Bichon Frise	10.90	13.01 (12.82–13.20)	251	Mesaticephalic
Border Collie	11.18	13.34 (13.15–13.53)	1289	Mesaticephalic
Border Terrier	11.88	14.12 (13.98–14.26)	516	Mesaticephalic
Boston Terrier	9.16	10.95 (10.78–11.12)	26	Brachycephalic
Boxer	9.14	10.88 (10.75–11.01)	345	Brachycephalic
Bull Terrier	9.71	11.61 (11.43–11.79)	125	Mesaticephalic
Bulldog	7.11	8.57 (8.36–8.78)	146	Brachycephalic
Cairn Terrier	11.49	13.66 (13.52–13.8)	145	Mesaticephalic
Cavalier King Charles Spaniel	9.77	11.64 (11.50–11.79)	637	Brachycephalic
Chihuahua	10.08	12.09 (11.85–12.33)	287	Brachycephalic
Chinese Crested	11.03	13.17 (12.97–13.37)	32	Mesaticephalic
Cockapoo	9.29	11.24 (10.93–11.55)	62	Mesaticephalic
Collie (Generic)	11.30	13.51 (13.30–13.72)	170	Dolichocephalic
Crossbreed	11.24	13.43 (13.23–13.62)	6355	Other
Dachshund	10.75	12.80 (12.53–13.07)	91	Dolichocephalic
Dachshund (Miniature)	11.15	13.23 (13.03–13.43)	105	Dolichocephalic
Dalmatian	10.36	12.39 (12.17–12.61)	123	Dolichocephalic
Dobermann	8.72	10.42 (10.19–10.65)	73	Dolichocephalic
Dogue De Bordeaux	5.57	6.70 (6.53–6.87)	37	Brachycephalic
English Setter	10.96	13.03 (12.88–13.18)	19	Dolichocephalic
Fox Terrier	11.13	13.40 (13.17–13.63)	23	Mesaticephalic
French Bulldog	7.54	9.08 (8.85–9.31)	200	Brachycephalic
German Pointer	10.25	12.23 (12.03–12.43)	61	Mesaticephalic
German Shepherd	9.67	11.47 (11.36–11.58)	726	Dolichocephalic
Great Dane	6.79	8.14 (7.96–8.32)	33	Dolichocephalic
Greyhound	10.79	13.01 (12.83–13.19)	298	Dolichocephalic
Hungarian Vizsla	9.83	11.71 (11.50–11.92)	56	Dolichocephalic
Husky (generic)	10.60	12.70 (12.55–12.85)	51	Dolichocephalic
Irish Setter	10.40	12.57 (12.30–12.84)	43	Dolichocephalic
Irish Terrier	11.25	13.36 (13.03–13.69)	17	Mesaticephalic
Italian Spinone	9.46	11.27 (10.99–11.55)	16	Dolichocephalic
Jack Russell Terrier	12.07	14.37 (14.24–14.50)	2151	Dolichocephalic
Japanese Akita Inu	8.30	9.94 (9.67–10.21)	42	Mesaticephalic
King Charles Spaniel	9.95	11.88 (11.67–12.09)	44	Brachycephalic
Labradoodle	10.91	12.95 (12.73–13.17)	135	Mesaticephalic
Lakeland Terrier	11.72	14.06 (13.82–14.30)	73	Mesaticephalic
Leonberger	5.95	7.14 (6.88–7.40)	12	Mesaticephalic
Lhasa Apso	10.50	12.56 (12.41–12.71)	345	Mesaticephalic
Lurcher	10.90	13.04 (12.82–13.26)	330	Dolichocephalic
Maltese	10.25	12.33 (12.06–12.61)	35	Mesaticephalic
Mastiff	7.66	9.20 (9.01–9.39)	40	Brachycephalic
Miniature Schnauzer	10.74	12.77 (12.63–12.91)	207	Mesaticephalic
Newfoundland	8.66	10.38 (10.19–10.57)	29	Mesaticephalic
Norfolk Terrier	11.43	13.61 (13.44–13.78)	24	Mesaticephalic
Old English Sheepdog	10.05	12.00 (11.82–12.18)	25	Mesaticephalic
Papillon	12.12	14.46 (14.26–14.67)	30	Mesaticephalic
Parson Russell Terrier	11.78	14.08 (13.86–14.30)	56	Dolichocephalic
Patterdale Terrier	11.55	13.81 (13.59–14.03)	191	Mesaticephalic
Pekingese	11.15	13.29 (13.12–13.47)	29	Brachycephalic
Pointer	10.17	12.16 (11.96–12.36)	45	Mesaticephalic
Pomeranian	10.28	12.33 (12.1–12.56)	41	Mesaticephalic
Poodle (generic)	11.35	13.53 (13.35–13.71)	84	Dolichocephalic
Poodle (Miniature)	11.76	14.02 (13.84–14.20)	77	Mesaticephalic
Poodle (Toy)	12.45	14.93 (14.65–15.21)	68	Mesaticephalic
Pug	8.92	10.73 (10.49–10.97)	151	Brachycephalic
Retriever (Flat Coated)	9.12	10.94 (10.73–11.15)	36	Mesaticephalic
Retriever (Generic)	11.02	13.10 (12.96–13.24)	90	Mesaticephalic
Retriever (Golden)	10.93	13.01 (12.85–13.17)	423	Mesaticephalic
Retriever (Labrador)	10.83	12.88 (12.74–13.02)	2545	Mesaticephalic
Rhodesian Ridgeback	10.04	11.97 (11.81–12.13)	45	Mesaticephalic
Rottweiler	8.10	9.69 (9.53–9.85)	231	Mesaticephalic
Saluki	10.12	12.20 (11.91–12.49)	18	Dolichocephalic
Samoyed	10.39	12.49 (12.25–12.73)	15	Mesaticephalic
Schnauzer	10.97	13.15 (12.95–13.35)	36	Dolichocephalic
Scottish Terrier	11.07	13.24 (13.00–13.48)	57	Mesaticephalic
Shar-Pei	9.31	10.99 (10.74–11.24)	74	Mesaticephalic
Shetland Sheepdog	11.65	13.86 (13.69–14.03)	73	Dolichocephalic
Shih Tzu	10.95	13.10 (12.91–13.29)	606	Brachycephalic
Siberian Husky	10.31	12.35 (12.16–12.54)	100	Dolichocephalic
Spaniel (American Cocker)	9.36	11.09 (10.79–11.39)	23	Brachycephalic
Spaniel (Cocker)	10.92	12.95 (12.78–13.12)	1241	Brachycephalic
Spaniel (English Springer)	10.83	12.81 (12.62–13.00)	154	Brachycephalic
Spaniel (Field)	10.28	12.19 (11.85–12.53)	13	Brachycephalic
Spaniel (Generic)	11.09	13.37 (13.06–13.68)	36	Brachycephalic
Spaniel (Springer)	11.13	13.41 (13.24–13.58)	883	Brachycephalic
Spaniel (Welsh Springer)	10.62	12.86 (12.55–13.17)	26	Brachycephalic
St. Bernard	6.66	7.98 (7.79–8.17)	13	Brachycephalic
Staffordshire Bull Terrier	10.41	12.25 (12.15–12.35)	1724	Mesaticephalic
Terrier (Generic)	11.08	13.22 (13.09–13.35)	171	Mesaticephalic
Tibetan Spaniel	11.03	13.16 (12.89–13.43)	13	Mesaticephalic
Tibetan Terrier	10.70	12.81 (12.53–13.09)	75	Mesaticephalic
Weimaraner	10.18	12.13 (11.92–12.34)	88	Dolichocephalic
Welsh Terrier	11.11	13.22 (12.83–13.61)	16	Mesaticephalic
West Highland White Terrier	11.45	13.67 (13.53–13.81)	896	Mesaticephalic
Whippet	10.73	12.86 (12.58–13.14)	195	Dolichocephalic
Yorkshire Terrier	11.82	14.01 (13.91–14.11)	797	Mesaticephalic

The breed-specific premature longevity thresholds is defined as 85% of the lower 95% confidence interval of the median age.

**Table 2:** Summary of median age at death, breed-specific premature longevity thresholds, total count (N), and Cephalic index for cat breeds within the dataset.

Breed	Premature threshold (years)	Median age (years)	n	Cephalic index
Abyssinian	12.69	13.25 (13.01–13.49)	16	Mesaticephalic
Bengal	10.93	15.52 (15.25–15.79)	116	Mesaticephalic
Birman	13.74	14.66 (14.43–14.89)	137	Mesaticephalic
Brazilian Shorthair	12.26	16.45 (16.13–16.77)	43	Mesaticephalic
British Shorthair	12.31	16.50 (16.24–16.77)	417	Brachycephalic
Burmese	13.80	9.84 (9.58–10.10)	144	Brachycephalic
Crossbreed	12.22	13.17 (12.86–13.48)	584	Dolichocephalic
Devon Rex	13.71	13.55 (13.30–13.80)	20	Brachycephalic
Domestic Long Hair	12.93	16.37 (16.17–16.57)	2132	Mesaticephalic
Domestic Medium Hair	11.72	15.47 (15.21–15.72)	574	Mesaticephalic
Domestic Short Hair	12.96	15.13 (14.93–15.33)	17318	Mesaticephalic
Maine Coon	10.87	14.11 (13.79–14.42)	188	Mesaticephalic
Norwegian Forest Cat	11.06	14.00 (13.74–14.26)	25	Mesaticephalic
Oriental Shorthair	8.14	14.01 (13.67–14.33)	16	Mesaticephalic
Persian	12.54	14.79 (14.48–15.09)	293	Mesaticephalic
Ragdoll	11.62	15.02 (14.76–15.28)	120	Dolichocephalic
Russian Blue	11.30	14.99 (14.77–15.21)	26	Dolichocephalic
Siamese	11.68	14.66 (14.37–14.95)	256	Mesaticephalic
Tonkinese	12.55	13.08 (12.79–13.37)	35	Mesaticephalic

The ‘breed-specific premature longevity thresholds’ is defined as 85% of the lower 95% confidence interval of the median age.

Tables 1 and 2 presents the bootstrapped median life expectancies for each breed found within our dataset across dogs and cats respectively. The defined breed-specific premature longevity thresholds, calculated as 0.85 times the lower confidence interval (CI) of their respective life expectancies is also present. We additionally categorise breeds based on the Cephalic index into the brachycephalic, mesaticephalic or dolichocephalic classifications. We observe in dogs the median age is highest for the mesaticephalic breeds at 13.15 years, followed by dolichocephalic at 12.71 years and the brachycephalic at 11.6 years. In cats, the inverse was observed. Brachycephalic cats had the longest median lifespan at 14.79 years, followed by mesaticephalic at 14.55 years and dolichocephalic at 14.00 years.

#### Causes of mortality

**Figure 1:**
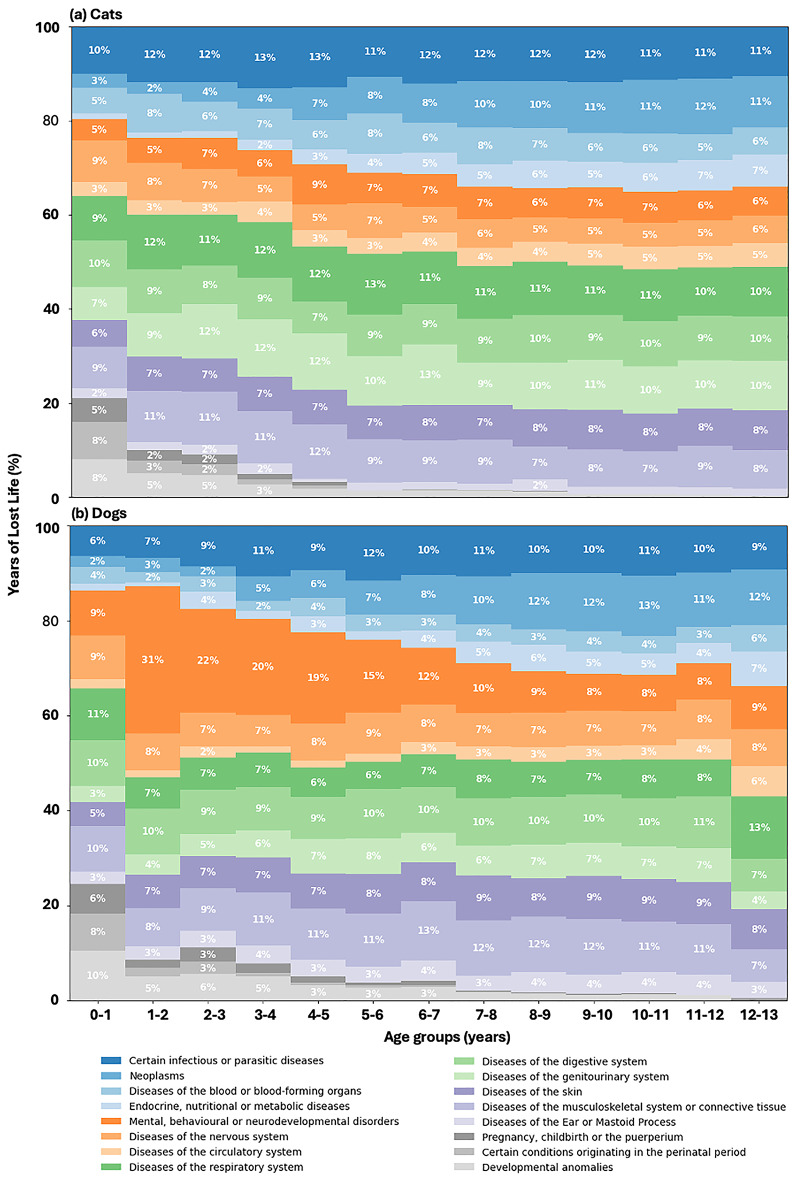
Proportional years of life lost (YLL) as a percentage due to assigned ICD-11 chapters at death across different age groups ranging from 0 to 13 years for (**a**) cats and (**b**) dogs.

We utilised ICD-11 chapter labels automatically assigned using PetBERT^26^ to analyse the causes of death among animals. For each ICD-11 chapter, we calculated the proportion of total years of lost life (YLL) attributed to that chapter within each age group. As depicted in Fig. 1, the results demonstrate the distribution of life-limiting conditions across different life stages in cats and dogs. For cats, *‘Neoplasms’* emerge as a significant cause of YLL, particularly in older age groups, reaching a peak of 12% in years 11–12. *‘Certain infectious or parasitic diseases’* are the leading cause of YLL from ages 0 to 5. As cats age, the impact of endocrine, nutritional, or metabolic diseases becomes more pronounced, with YLL increasing from less than 5% in ages 0–7 to 7% beyond that age range. *‘Diseases of the genitourinary, digestive, and respiratory systems’* consistently manifest across all age groups, with respiratory diseases being notably high in the youngest and oldest cats. In dogs, *‘Mental, behavioural or neurodevelopmental disorders’* are the predominant cause of YLL between years 1 and 2, accounting for 31%, and remain the leading cause until the age group of 6–7 years. *‘Neoplasms’* become increasingly significant as dogs age, starting at 2–3% YLL in ages 0-3 and rising to 12% by ages 12–13. Respiratory conditions initially account for 11% YLL, stabilise at 7–8% during middle age, and increase again to 13% in dogs aged 12–13.

An additional examination of YLL data across various dog breed types-brachycephalic, mesaticephalic, and dolichocephalic -unveiled notable disparities in the primary causes of mortality among these groups. The findings can be found within the supplementary materials in Fig. 1. We continued the use of the ICD-11 classification to categorise the causes of YLL, with the results presented in two-year intervals due to the reduced sample sizes in the breed-specific analyses. For brachycephalic breeds, *‘Developmental anomalies’* emerged as the most significant cause of YLL in the 0–2 age group, accounting for 14.0% of the YLL. This was notably higher than mesaticephalic and dolichocephalic breeds, which reported *‘Developmental anomalies’* at 7.0%. Instead, *‘Mental, behavioural, or neurodevelopmental disorders’* was the predominant cause of YLL in these latter breeds, with 20.0% for mesaticephalic and 19.0% for dolichocephalic breeds. Across all breeds, *‘Neoplasms’* exhibited a marked increase in YLL proportion with age. The YLL due to ‘Neoplasms’ peaked at 12% for both brachycephalic and mesaticephalic breeds in the older age groups. In contrast, dolichocephalic breeds showed a significantly higher YLL proportion due to *‘Neoplasms’*, reaching 17.0%. *‘Diseases of the musculoskeletal system or connective tissue’* were present across all breed groups but were most prominent in dolichocephalic breeds. In the 4–6 age group, the YLL proportion was 18.0%, and it remained the highest among the breeds at 14.0% in the 10–12 age group. This compares to 10.0% for brachycephalic and mesaticephalic breeds in the same age group. *‘Endocrine, nutritional, or metabolic diseases’* constituted a minor cause of YLL in brachycephalic and mesaticephalic breeds, each accounting for 3.0–4.0% of YLL. However, in dolichocephalic breeds, the YLL proportion for these diseases doubled, reaching 8.0%. *‘Diseases of the respiratory system’* showed a higher average YLL proportion in brachycephalic breeds, averaging 9% across all age groups. This was compared to 7.6% in mesaticephalic breeds and 6.5% in dolichocephalic breeds, indicating a breed-specific predisposition.

### Risk factors for premature mortality

Using an initial univariate mixed-effect logistic regression model, we evaluated odds ratios for the risk factors associated with premature mortality in dogs and cats. Given certain demographic and phenotypic characteristics, our objective was to determine the likelihood of death before the breed-specific premature longevity thresholds. After applying our selection criteria, which included a likelihood ratio test (LRT chi-squared test) with a p-value threshold of $\le$ 0.2, we found that neuter status and urban-rural scoring did not meet the inclusion criteria for the multivariable analysis. The results of the multivariable logistic regression for dogs and cats using brachycephalic as a binary variable is presented in Tables 3 and 4, respectively. Additionally, the individual cephalic index can be found in supplementary materials tables 1 and 2 for dogs and cats, respectively.

**Table 3:** Multivariable logistic regression analysis identifying risk factors for premature mortality in dogs, comparing cases (death below breed-specific longevity threshold) and controls (death after threshold).

Variable	Level	Case (%)	Control (%)	OR (95% CI)	Beta	SE	P
	(Intercept)	–	–	0.16 (0.15–0.18)	− 1.83	0.05	<0.001
Sex	Male	3115 (51%)	10985 (50%)	–	–	–	–
	Female	2939 (49%)	11120 (50%)	0.96 (0.90–1.03)	− 0.04	0.03	<0.001
Index of multiple deprivation (IMD)	1	1118 (18%)	3247 (15%)	–	–	–	–
	2	1235 (20%)	4042 (18%)	0.90 (0.80–1.00)	− 0.11	0.06	<0.001
	3	1424 (24%)	5028 (23%)	0.85 (0.77–0.95)	− 0.16	0.06	<0.001
	4	1274 (21%)	5198 (24%)	0.77 (0.69–0.86)	− 0.26	0.06	<0.001
	5	1003 (17%)	4590 (21%)	0.62 (0.55–0.70)	− 0.48	0.06	<0.001
Brachycephalic	No	5296 (87%)	19934 (90%)	–	–	–	–
	Yes	758 (13%)	2171 (10%)	1.19 (1.07–1.32)	0.17	0.05	<0.001
Certain infectious or parasitic diseases	No	5173 (85%)	20955 (95%)	–	–	–	–
	Yes	881 (15%)	1150 (5%)	1.36 (1.19–1.54)	0.30	0.07	<0.001
Neoplasms	No	5142 (85%)	21096 (95%)	–	–	–	–
	Yes	912 (15%)	1009 (5%)	2.52 (2.25–2.83)	0.93	0.06	<0.001
Diseases of the blood or blood forming organs	No	5856 (97%)	22087 (99%)	–	–	–	–
	Yes	198 (3%)	18 (1%)	21.83 (13.07–36.46)	3.08	0.26	<0.001
Diseases of the Immune system	No	5822 (96%)	21751 (98%)	–	–	–	–
	Yes	232 (4%)	354 (2%)	1.61 (1.30–1.99)	0.48	0.11	<0.001
Endocrine nutritional or metabolic diseases	No	5749 (95%)	21676 (98%)	–	–	–	–
	Yes	305 (5%)	429 (2%)	1.56 (1.31–1.87)	0.45	0.09	<0.001
Mental behavioural or neurodevelopmental disorders	No	5172 (85%)	21529 (97%)	–	–	–	–
	Yes	882 (15%)	576 (3%)	5.69 (5.04–6.43)	1.74	0.06	<0.001
Diseases of the nervous system	No	5511 (91%)	21922 (99%)	–	–	–	–
	Yes	543 (9%)	183 (1%)	7.01 (5.79–8.48)	1.95	0.10	<0.001
Diseases of the visual system	No	5502 (91%)	21485 (97%)	–	–	–	–
	Yes	552 (9%)	620 (3%)	1.41 (1.22–1.63)	0.34	0.08	<0.001
Diseases of the ear or mastoid process	No	5697 (94%)	21555 (98%)	–	–	–	–
	Yes	357 (6%)	550 (2%)	1.40 (1.19–1.66)	0.34	0.09	<0.001
Diseases of the circulatory system	No	5807 (96%)	21892 (99%)	–	–	–	–
	Yes	247 (4%)	213 (1%)	2.15 (1.72–2.69)	0.77	0.11	<0.001
Diseases of the respiratory system	No	5497 (91%)	21838 (99%)	–	–	–	–
	Yes	557 (9%)	267 (1%)	5.60 (4.73–6.64)	1.72	0.09	<0.001
Diseases of the digestive system	No	5337 (88%)	21623 (98%)	–	–	–	–
	Yes	717 (12%)	482 (2%)	4.42 (3.84–5.08)	1.49	0.07	<0.001
Diseases of the skin	No	5240 (87%)	20977 (95%)	–	–	–	–
	Yes	814 (13%)	1128 (5%)	1.58 (1.38–1.80)	0.46	0.07	<0.001
Diseases of the musculoskeletal system or connective tissue	No	5047 (83%)	21171 (96%)	–	–	–	–
	Yes	1007 (17%)	934 (4%)	2.40 (2.11–2.73)	0.87	0.07	<0.001
Diseases of the genitourinary system	No	5591 (92%)	21844 (99%)	–	–	–	–
	Yes	463 (8%)	261 (1%)	4.38 (3.66–5.24)	1.48	0.09	<0.001
Pregnancy childbirth or the puerperium	No	5991 (99%)	22091 (99%)	–	–	–	–
	Yes	63 (1%)	14 (1%)	5.91 (2.97–11.76)	1.78	0.35	<0.001
Certain conditions originating in the perinatal period	No	6005 (99%)	22102 (99%)	–	–	–	–
	Yes	49 (1%)	3 (1%)	21.62 (6.20–75.41)	3.07	0.64	<0.001
Developmental anomalies	No	5890 (97%)	22025 (99%)	–	–	–	–
	Yes	164 (3%)	80 (1%)	2.03 (1.47–2.80)	0.71	0.17	<0.001
Injury poisoning or certain other consequences of external causes	No	5403 (89%)	21308 (96%)	–	–	–	–
	Yes	651 (11%)	797 (4%)	1.85 (1.60–2.14)	0.62	0.07	<0.001

*OR* odds ratio, *CI* lower and upper 95% confidence interval, *SE* standard error.

**Table 4:** Multivariable logistic regression analysis identifying risk factors for premature mortality in cats, comparing cases (death below breed-specific longevity threshold) and controls (death after threshold).

Variable	Level	Case (%)	Control (%)	OR (95% CI)	Beta	SE	P
	(Intercept)	–	–	0.26 (0.23–0.29)	− 1.36	0.06	0.001
Sex	Male	3582 (53%)	9738 (57%)		–	–	–
	Female	3226 (47%)	7460 (43%)	0.72 (0.67–0.77)	− 0.33	0.04	0.001
Index of multiple deprivation (IMD)	1	1128 (17%)	1845 (11%)		–	–	–
	2	1288 (19%)	2778 (16%)	0.78 (0.69–0.89)	− 0.25	0.07	0.001
	3	1505 (22%)	3768 (22%)	0.68 (0.6–0.77)	− 0.38	0.06	0.001
	4	1534 (23%)	4160 (24%)	0.65 (0.57–0.73)	− 0.44	0.06	0.001
	5	1353 (20%)	4647 (27%)	0.52 (0.46–0.60)	− 0.65	0.07	0.001
Brachycephalic	No	1686 (20%)	4919 (27%)		–	–	–
	Yes	5837 (86%)	14994 (87%)	1.00 (0.82–1.22)	0.00	0.10	1.000
Certain infectious or parasitic diseases	No	5400 (79%)	16390 (95%)		–	–	–
	Yes	1408 (21%)	808 (5%)	2.10 (1.84–2.40)	0.74	0.07	0.001
Neoplasms	No	5914 (87%)	17074 (99%)		–	–	–
	Yes	894 (13%)	124 (1%)	15.53 (12.62–19.13)	2.74	0.11	0.001
Diseases of the blood or blood forming organs	No	6286 (92%)	17170 (99%)		–	–	–
	Yes	522 (8%)	28 (1%)	17.31 (11.46–26.16)	2.85	0.21	0.001
Diseases of the Immune system	No	6597 (97%)	17021 (99%)		–	–	–
	Yes	211 (3%)	177 (1%)	1.07 (0.81–1.41)	0.06	0.14	0.660
Endocrine nutritional or metabolic diseases	No	6301 (93%)	16870 (98%)		–	–	–
	Yes	507 (7%)	328 (2%)	1.97 (1.64–2.37)	0.68	0.09	0.001
Mental behavioural or neurodevelopmental disorders	No	6165 (91%)	16862 (98%)		–	–	–
	Yes	643 (9%)	336 (2%)	2.13 (1.79–2.53)	0.76	0.09	0.001
Diseases of the nervous system	No	6265 (92%)	17159 (99%)		–	–	–
	Yes	543 (8%)	39 (1%)	19.18 (13.47–27.29)	2.95	0.18	0.001
Diseases of the visual system	No	6061 (89%)	16976 (99%)		–	–	–
	Yes	747 (89%)	222 (1%)	3.05 (2.53–3.69)	1.12	0.10	0.001
Diseases of the ear or mastoid process	No	6655 (98%)	17116 (99%)		–	–	–
	Yes	153 (2%)	82 (1%)	1.98 (1.41–2.77)	0.68	0.17	0.001
Diseases of the circulatory system	No	6367 (94%)	17040 (99%)		–	–	–
	Yes	441 (6%)	158 (1%)	3.26 (2.59–4.12)	1.18	0.12	0.001
Diseases of the respiratory system	No	5719 (84%)	17020 (99%)		–	–	–
	Yes	1089 (16%)	178 (1%)	9.50 (7.93–11.39)	2.25	0.09	0.001
Diseases of the digestive system	No	5888 (86%)	16909 (98%)		–	–	–
	Yes	920 (14%)	289 (2%)	4.69 (3.98–5.53)	1.55	0.08	0.001
Diseases of the skin	No	5929 (87%)	16729 (97%)		–	–	–
	Yes	879 (13%)	469 (3%)	2.01 (1.68–2.41)	0.70	0.09	0.001
Diseases of the musculoskeletal system or connective tissue	No	5971 (88%)	17001 (99%)		-	-	-
	Yes	837 (12%)	197 (1%)	4.77 (3.89–5.85)	1.56	0.10	0.001
Diseases of the genitourinary system	No	5771 (85%)	16979 (99%)		–	–	–
	Yes	1037 (15%)	219 (1%)	7.71 (6.49–9.14)	2.04	0.09	0.001
Pregnancy childbirth or the puerperium	No	6759 (99%)	17197 (99%)		–	–	–
	Yes	49 (1%)	1 (1%)	11.18 (1.25–99.83)	2.41	1.12	0.030
Certain conditions originating in the perinatal period	No	6735 (99%)	17197 (99%)		–	–	–
	Yes	73 (1%)	1 (1%)	59.42 (7.65–461.71)	4.09	1.05	0.001
Developmental anomalies	No	6665 (99%)	17188 (99%)		–	–	–
	Yes	143 (1%)	10 (1%)	10.18 (4.89–21.18)	2.32	0.37	0.001
Injury poisoning or certain other consequences of external causes	No	6185 (91%)	16888 (98%)		–	–	–
	Yes	623 (9%)	310 (2%)	2.39 (1.99–2.88)	0.87	0.09	0.001

*OR* odds ratio, *CI* lower and upper 95% confidence interval, *SE* standard error.

Our findings indicate a significant decrease in the risk of premature mortality as deprivation levels decrease (IMD 5: OR for dogs = 0.62, p < 0.001; cats = 0.52, p < 0.001) relative to the most deprived category (IMD 1). This trend was consistent across both species. Analysis of ICD-11 disease groups revealed substantial variations in risk. For instance, *’developmental anomalies’*, more common in younger animals, were associated with a 10.18-fold increase in premature mortality in cats and a 2.03-fold increase in dogs. *’Conditions originating in the perinatal period’* posed the highest risk, with a 59.42-fold increase in cats and a 21.62-fold increase in dogs. Moreover, *‘Diseases of the blood and blood-forming organs’* exhibited particularly high odds ratios for premature mortality in both species (cats: 17.31; dogs: 21.83). Similarly, ’Diseases of the nervous system’ had high-risk ratios (cats: 19.18; dogs: 7.01). A slight protective effect was observed for females, with odds ratios of 0.72 in cats and 0.97 in dogs. Certain conditions displayed species-specific impacts. For instance, behavioural issues, a known risk factor for euthanasia in dogs, had an odds ratio of 5.69, compared to 2.13 in cats, highlighting notable differences in the impact of behavioural factors between species. Additionally, brachycephalic dogs were associated with a 1.19-fold increase in the odds of premature mortality, while the same analysis in cats showed no significant effect.

## Discussion

Unlike human medicine, veterinary medicine does not have a death register, leaving the frequency and cause of companion animal death poorly quantified. Previous works have aimed to use insurance data to achieve this goal^28,29^. However, such methods are inherently biased such as towards certain breeds^30^, incomplete data due to limitations imposed by the insurance companies^31^, or insurance claims only being made in more severe disease cases^32^. Identifying the risk factors for premature death in companion animals from first-opinion clinical narratives offers new insights into opportunities for targeted healthcare measures. There are challenges in maximising first-opinion clinical narrative’s utility; for example, their free-text nature and absence of standardised disease coding systems make extracting meaningful clinical events from the narratives difficult^33^.

The observed inequalities in companion animal life longevity reveal a significant correlation between socioeconomic status and premature mortality rates. Data indicates a clear trend: as the index of multiple deprivations (IMD) scores decrease, indicating less deprivation, the probability of premature mortality also decreases for both cats (OR = 0.52, p < 0.001) and dogs (OR = 0.62, p < 0.001). This disparity underscores the social inequalities mirrored in human health outcomes^34,35^. Several factors likely contribute to this increased mortality in deprived areas. Pets in wealthier households often have better and more frequent access to veterinary care, which includes the ability to afford advanced treatments and diagnostics^36^. Furthermore, wealthier owners are more likely to afford comprehensive health insurance for their pets, reducing the financial barrier to accessing necessary medical care. Across the SAVSNET dataset, the highest insurance rate at 40.3% is observed in the least deprived (IMD5), steadily decreasing to 29.6% in the most deprived quintile (IMD1). A previous study has looked at the link between euthanasia and financial stress and revealed that owners would be willing to increase the amount spent at a given visitation if they had pet health insurance^37^. Discussions around being unable to afford available treatment and thus opting for euthanasia instead were frequently observed. Other factors, such as the increased quality of the animal’s diet brought about by a more affluent household, also likely play a role^38^. These findings point to the multi-faceted nature of the issue, suggesting that addressing these inequalities requires a holistic approach. Further research is necessary to explore these dimensions and develop strategies to mitigate the impact of socioeconomic disparities on companion animal health.

Our analysis reinforces the established understanding that brachycephalic breeds generally experience poorer health outcomes compared to their non-brachycephalic counterparts^15,17^. Specifically, we found a higher number of years of life lost (YLL) due to developmental anomalies in brachycephalic breeds during the first two years of life compared to mesaticephalic and dolichocephalic breeds. This is consistent with previous studies suggesting issues related to excess soft tissue, aberrant conchal growth, and obstruction of the nasal passage^20,39,40^. Furthermore, the average lifespan of brachycephalic breeds was reduced to 10.95 years, compared to 12.51 years for mesaticephalic breeds and 12.46 years for dolichocephalic breeds, corroborating prior research indicating shorter lifespans in this category^17,41^. Respiratory disease also contributed to a higher average YLL proportion in brachycephalic breeds, averaging 9% across all age groups, this can be described through the impacts of Brachycephalic Obstructive Airway Syndrome (BOAS) and pneumonia^19,20^. However, certain conditions, such as *‘Neoplasms‘*, exhibited a similar propensity across all breed types. The prevalence of *‘Neoplasms‘* increased from below 2–3% before age 3–12% by ages 11–12, mirroring trends observed across both species and supporting the notion that older mammals are generally more prone to neoplasias.

Of note within our analysis is the significant years of lost life for young dogs, namely aged 1–2 years, as a result of behavioural disorders. Behavioural disorders have previously been discussed as a leading cause of negative welfare outcomes such as euthanasia, with one study finding that behaviour was the fourth most common reason for euthanasia, accounting for 7.3% of euthanasia deaths in dogs^42^. Our results suggest a significant increased risk (OR 5.69, p < 0.001) of premature mortality in dogs with behavioural disorders compared to dogs without, which is supported by previous literature that suggests age is a risk factor for the development of problematic behaviour, with younger dogs (particularly adolescent dogs under three years old), potentially more prone to problematic behaviours increasing their risk of euthanasia or relinquishment. Existing data from a UK-based study, also using primary-care consult data, showed that around one-third of deaths in dogs under three years are ascribed to behavioural issues, and of these deaths, 75% are from euthanasia^43^. Similarly, an Australian-based study revealed that 29.7% of dogs under primary care that died at three years of age or under had deaths attributed to at least one problematic behaviour^44^. Many studies suggest aggression, in particular, is commonly related to premature death in younger dogs and is frequently reported as the most common cause of behavioural euthanasia^43–45^. As well as euthanasia, problematic behaviour has previously been discussed as a leading cause of relinquishment of young dogs to shelters^46^, as well as a factor in unsuccessful adoptions from shelters^47^. A study by Clark et al. also found that behavioural problems accounted for 65.6% of dogs euthanised in UK rehoming centres in 2009, which could suggest that many dogs may also have been relinquished due to behavioural issues prior to their euthanasia^48^. With the evidence growing around the higher prevalence of behaviour problems and subsequent relinquishment and euthanasia in younger dogs, further investigation is warranted to determine associated factors behind this and thus implement preventative measures to reduce the likelihood of problematic behaviours firstly developing and further resulting in euthanasia or relinquishment where they do occur.

The ICD-11 syndromic labels offer only a broad categorisation of events in the narratives, not specific causes of animal deaths. Further research is needed to extract detailed causal information. The quality and completeness of free-text consultation notes varied significantly among attending clinicians, introducing inconsistency in data quality. These factors collectively limited our ability to extract comprehensive and accurate information from all cases. While our previous work demonstrated our model achieving an 83% F1 score accuracy for the ICD-11 assignment, it suggests that a small proportion of records will be incorrectly assigned. Additionally, our approach of considering animals with multiple ICD-11 labels as having each syndrome equally contributing to their death is a simplification that may not accurately reflect the true clinical outcome. Future research should focus on developing more sophisticated methods to extract true causes of death, moving beyond the distributed approach employed in this study. Our study also categorised all mixed-breed dogs as a single group within our breed-specific mean lifespan calculations. This broad categorisation encompasses a diverse range of phenotypic features, which may include brachycephalic traits not accounted for in the cephalic index risk factor analysis. While we acknowledge the well-documented relationship between size and lifespan in dogs, our analysis did not apply size-based groupings of mixed-breed dogs. Given that mixed-breed dogs comprise approximately 20% of all deceased dogs in the dataset, we recognise that size-based classifications might have influenced the number of cases and controls, potentially affecting the calculated ORs. Nonetheless, we believe that our findings remain robust, as the inclusion of this large subgroup reflects a substantial proportion of the population, and the overall trends observed in the data still hold. However, future research could explore more granular categorisations of mixed-breed dogs in assessing potential impacts on lifespan-related outcomes. Although we made efforts to minimise false positives and false negatives in identifying deceased animals, some inaccuracies likely remain within the dataset. This omission likely under-represents the number of animals that died without euthanasia. Lastly, the SAVSNET network only partially represents veterinary consultations and the companion animal population across the UK.

In conclusion, this study on premature mortality in dogs and cats in the UK has provided valuable insights into the complex interplay of factors affecting companion animal longevity. By leveraging LLM methodology, we have enabled the analysis of a substantial dataset of deceased animals; we have uncovered critical patterns in years of lost life across various age groups and identified key risk factors associated with premature mortality. The significant impact of behavioural conditions on premature euthanasia in dogs, particularly in younger age brackets, highlights an urgent need for improved behavioural support and intervention strategies. Furthermore, our investigation into premature mortality risk factors reveals a significant correlation with socioeconomic status, underscoring how the impact of social inequalities extends to companion animal health. This research identifies areas needing urgent attention and underscores the importance of continued investigation into the socioeconomic disparities affecting animal health outcomes. Our findings advocate for targeted interventions and emphasise the necessity for enhanced research efforts to address and mitigate these inequities, paving the way for more equitable health prospects for companion animals across the UK.

## Methods

### Dataset

Electronic health records have been collected since March 2014 by SAVSNET, the Small Animal Veterinary Surveillance Network, comprising a sentinel network of 253 volunteer veterinary practices across the United Kingdom. A full description of SAVSNET has been presented elsewhere^9^. In summary, veterinary practices with compatible practice management software with the SAVSNET data exchange are recruited based on convenience. Within these participating practices, data is collected from each booked consultation (where an appointment has been made to see a veterinary practitioner or nurse). All owners attending a participating practice are informed of the data collection process and are given the opportunity to opt-out during their consultation. At this point, data is not sent to the SAVSNET data exchange and, therefore, is not utilised in any research. Pet owners are provided with informed consent that their data could be used for research purposes. Data is collected on a consultation-by-consultation basis and includes information such as species, breed, sex, neuter status, age, owner’s postcode, insurance and microchipping status and, crucially to this study, a free-text clinical narrative outlining the events that occurred within that consultation. To support the current study, high-level syndromic labels corresponding to the chapters of the International Classification of Diseases 11 (ICD-11) were appended to each consultation, building on established methodologies. These labels and associated conditions adhere to the broad categorisation framework outlined by the World Health Organisation (WHO)^27^. This method leveraged PetBERT, a large language model that was further pre-trained on the SAVSNET corpus and subsequently fine-tuned to function as a multi-label classifier covering the ICD-11’s high-level categories. The original study^26^ provides a comprehensive explanation of this approach. Sensitive information, including personal identifiers, was cleaned from the dataset prior to analysis. Additionally, all data discussed within this paper is at a population level; therefore, no specific individual will be discussed. SAVSNET has ethical approval from the University of Liverpool Research Ethics Committee (RETH000964). We hereby confirm that all experiments were conducted in strict compliance with the Research Ethics Committee.

### Data extraction

Narratives mentioning death or euthanasia were identified using a generalised Python regular expression (regex) to screen for terms such as “euthanasia”, “put to sleep (PTS)”, and “died”. The final regex is outlined below. The generalised dataset was then randomly sampled for 250 suspected potential death/euthanasia cases. These were then manually read to verify that they met the case definition of a “declaration of death”. Common false positives included discussions related to future euthanasia events or euthanasia mentioned as advisory by the attending practitioner. Unless the euthanasia event occurred in the same consultation, these records were not annotated as cases. Adapting the works of Yalniz et. al., a semi-supervised teacher-student model approach was employed, where a small subset of manually annotated records was used to train a small binary sequence classification model^49^. The resultant model from this task was then used against the entire dataset to extract animals identified as a case, a random sample of 200 records was passed to a practising clinician to verify that the performance of the extraction method was sufficient enough to continue the study.

*euth|dead|died|pts|put to sleep|pento|doa|crem|burial|bury|qol|quality|ashes|scatter|casket*

The cephalic index for each breed was appended as appropriate. These labels were derived from various sources^17,50,51^. Animals identified as crossbreeds were labeled as ’other’ due to insufficient cephalic data.

### Defining premature mortality

To define breed-specific premature longevity thresholds for each breed within our dataset, we employed a bootstrapping method to estimate the median age at death for each breed. Specifically, we generated 10,000 new datasets through random sampling with replacement from the original dataset. For each new dataset, we calculated the median age at death for each breed and established a 95% confidence interval. Below 85% of the lower bound of this confidence interval served as the threshold for identifying premature mortality. To ensure robustness in our findings, we included only breeds with at least ten observations in both premature and expected death categories. Using a breed-specific 95% confidence interval, we accounted for the variances in life expectancy inherent to different breeds. An animal whose age at death was below the lower bound of its breed’s confidence interval was classified as having died prematurely.

### Premature mortality and years of lost life (YLL)

To analyse the causes of death among animals, we leveraged ICD-11 chapter labels automatically assigned using PetBERT^26^. In some cases, the final ’death’ record did not provide precise details regarding the cause of death. We extended our analysis to include consultations up to six months before the recorded death to address this. Any ICD-11 syndromic label present during this period was considered a potential indicator of the cause of death. We calculated the YLL for each age group (ages 1 to 14) using the following equation:

$$\begin{aligned} YLL = \sum _{i=1}^{n} (L_{i} - A_{i}) \end{aligned}$$

where $L_{i}$ is the estimated breed-specific premature longevity thresholds produced above for a given animal for the age group and $A_{i}$ is the age at death for each individual $i$. This computation was performed for each ICD-11 chapter to determine the YLL attributable to different causes of death. Records maybe annotated with multiple syndromes and therefore each will be counted independently. To quantify the proportions of lost life, we summed the total number of years lost for each age group due to death linked to each ICD-11 chapter and divided these by the total number of years of lost life for the same age group. This approach allowed us to understand the distributions and impacts of various causes of death across different age groups and disease classifications.

### Premature mortality risk factor analysis

We established the breed-specific premature longevity thresholds to investigate risk factors associated with premature death. Animals that died before this age were classified as experiencing premature death, while those living beyond this age were considered to have died ‘as expected’. We implemented a data truncation approach to mitigate survivorship bias and ensure a fair comparison between animals. Regardless of their actual lifespan, we only considered clinical events and data points occurring before the breed-specific premature longevity thresholds for all animals in the dataset. This approach effectively creates a standardised observation window for all animals in the study. Using this prepared dataset, we employed an initial univariate mixed-effect logistic regression model using case-control status as a binary dependent variable to identify potential risk factors that may increase the odds of an animal dying before their breed-specific longevity threshold. Animals identified as having died prematurely were categorised as cases, while controls were animals whose deaths occurred beyond the breed-specific premature threshold age. Each explanatory variable was analysed individually, and the fit compared to a null model was assessed using the likelihood ratio test (LRT chi-squared test), incorporating practice as a random effect to account for potential clustering. An initial multivariable logistic regression model was constructed by including only those explanatory variables that demonstrated an LRT p-value $\le$ 0.2 compared to the null model. This preliminary model was then refined through a backward selection process to achieve the best model fit characterised by the lowest possible Akaike information criterion (AIC). The final multivariable model was analysed for multicollinearity by calculating the variance inflation factor (VIF), confirming that multicollinearity was absent.

## Data Availability

The datasets analysed during the current study are not publicly available due to issues surrounding owner confidentiality. Reasonable requests can be made to the SAVSNET Data Access and Publication Panel (savsnet@liverpool.ac.uk) for researchers who meet the criteria for access to confidential data.
